# Current Psychopathological Symptoms in Children and Adolescents Who Suffered Different Forms of Maltreatment

**DOI:** 10.1155/2016/8654169

**Published:** 2016-08-04

**Authors:** Paola De Rose, Fortunata Salvaguardia, Paola Bergonzini, Flavia Cirillo, Francesco Demaria, Maria Pia Casini, Deny Menghini, Stefano Vicari

**Affiliations:** Child Neuropsychiatry Unit, Neuroscience Department, “Children's Hospital Bambino Gesù”, Piazza Sant'Onofrio 4, 00165 Rome, Italy

## Abstract

The aim of the present study is to evaluate the current psychopathological problems of different forms associated with maltreatment on children's and adolescents' mental health. Ninety-five females and ninety males with a mean age of 8.8 years who have suffered in the last six months different forms of abuse (physical, sexual, and emotional) and neglect were included in the study. The current reaction to trauma as directly observed by clinical instruments was examined. Differences in gender, age at the time of medical examination, familial psychiatric disorders, neuropsychiatric status, and type of maltreatment were also taken into account. Results documented that 95.1% of abused children and adolescents developed a psychiatric disorder or a subclinical form of a Posttraumatic Stress Disorder (PTSD). Moreover, our data demonstrate a role for gender, age, and familial psychiatric comorbidity in the current psychopathological problems associated with maltreatment. Overall, our findings can help clinicians make a diagnosis and provide efficient treatment and prevention strategies for child maltreatment and abuse.

## 1. Introduction

The World Health Organization (WHO, World Report on Child Injury Prevention) defines children maltreatment as “all forms of physical and/or emotional maltreatment, sexual abuse, neglect or negligent treatment or commercial or other exploitation, resulting in actual or potential harm to the child's health, survival, development or dignity in the context of a relationship of responsibility, trust or power” [1]. Four types of child maltreatment can be distinguished: physical abuse, defined as the intentional use of physical force against a child that results in (or has a high likelihood of resulting in) harm for the child's health, survival, development, or dignity (it includes hitting, beating, kicking, shaking, biting, strangling, scalding, burning, poisoning, and suffocating); sexual abuse, defined as the involvement of a child in sexual activity that he or she does not fully comprehend or is unable to give informed consent to or for which the child is not developmentally prepared or else that violates the laws or social taboos of society; emotional and psychological abuse, which involves both isolated incidents, as well as a pattern of failure over time on the part of a parent or caregiver to provide a developmentally appropriate and supportive environment (it includes the restriction of movement; patterns of belittling, blaming, threatening, frightening, discriminating against, or ridiculing; and other nonphysical forms of rejection or hostile treatment); neglect, which includes both isolated incidents, as well as a pattern of failure over time on the part of a parent or another family member to provide for the development and well-being of the child in one or more areas such as health, education, emotional development, nutrition, shelter, and safe living conditions [1].

The extent of child abuse varies from 3 to 6 children out of 1000 (WHO, 2008) and it represents a primary risk factor for an individual's mental health [2, 3]. Evidence has been collected in support of the relationship between child maltreatment and the development of a variety of short-term and long-term psychological and behavioral problems, psychiatric and somatic disorders, and neurocognitive deficits [4–8]. A strict association has been demonstrated between child maltreatment and the outset of externalizing mental health problems (i.e., ADHD, conduct disorder, and oppositional-defiant disorder), internalizing mental health problems (i.e., anxiety, depression, and panic disorder), autism, Posttraumatic Stress Disorder (PTSD), and antisocial personality [9–13]. Specifically, Rogosch et al. [12] reported that child maltreatment prior to age 8 significantly predicted both externalizing and internalizing problems and low social competences, which persist even into earlier school-age (7–9 years) to later adolescence (15–18 years). Moreover, exposure to maltreatment during childhood is related to poor school performances [11, 14, 15] and to deficits in cognitive functions, such as language, memory, attention, and executive functions [16–18]. Maltreated children may also exhibit deficits on emotion regulation, attachment organization, autonomous self-development, peer relationship functioning, adaptation to school, adolescent autonomy, romantic relationship, and identifying formation [12, 19–21].

The majority of the above reported studies were retrospective and results were based on self-report questionnaires collected from adults. Current psychopathological problems in maltreated children have been only partially investigated especially through the use of questionnaires. Reviewed studies on maltreated youth documented PTSD in 21–50% of sexually abused children and in up to 50% of physically abused ones [22–24]. In sexually abused children a substantial effect size regarding outcome toward PTSD was found, especially for reexperiencing symptoms [25]. Moreover, one-third to one-half of neglected children had PTSD symptoms and also in emotionally abused children PTSD symptomatology was extensively found [26]. PTSD is especially linked with longer duration of maltreatment, repetitive and abusive nature of the stressor, threat or force, feelings of guilt, exaggerated startle response, and perception that one has been victimized [7, 27–32].

Furthermore, psychopathological criteria in children derived from diagnosis for adults (DSM-IV-TR) [33], especially for PTSD and they may not accurately reflect responses to trauma in children.

The present study was aimed at deeply investigating current psychopathological problems of child abuse and neglect on children's and adolescents' mental health and development. According to the guideline's recommendations of the Italian Child and Adolescent Neuropsychiatry Society (SINPIA, 2007) we evaluated the presence of psychiatric disorders using semistructured interviews, questionnaires, structured behavioral observations, and parent development interviews. Moreover, we are interested at investigating whether clinical criteria for psychopathological diagnosis developed for adults are useful to capture symptoms in children who suffered different forms of maltreatment. In order to analyze the role of protective and risk factors on the development of child psychopathology, we explored the individual characteristics (i.e., child gender and age at time of maltreatment), the forms of maltreatment, and the familial characteristics (presence/absence of a first- or second-degree relative with psychiatric disorder). The prevalence of different forms of maltreatment in each and between age groups, different psychiatric diagnosis in each and between age groups, and different forms of maltreatment and of psychiatric diagnosis in males and in females was evaluated.

## 2. Materials and Methods

### 2.1. Participants

The sample was comprised of consecutive youth, ages 2–18 years, who were evaluated at Child Neuropsychiatric Unit, Neuroscience Department, the Children's Hospital Bambino Gesù between January 2011 and March 2012 in order to assess mental status in the context of suspected abuse in the previous six months. Our Child Neuropsychiatric Unit has developed child abuse-assessment teams of neuropsychiatrists and psychologists who specialize in the assessment of suspected victims of child abuse by using specific protocol about maltreatment, as required by hospital procedure. Our hospital has the most available expertise in the region in the emergency evaluation of suspected child abuse. The children arrived at the hospital in different paths: spontaneously (41%), through the Emergency Ward or the Pediatric Hospital Unit (36%), based on indications of the social services (21%) and on the courts (2%). Different characteristics, including population (gender, age at the time of medical examination, family history of psychiatric disorders, cognitive development, and neuropsychiatric status) and maltreatment (kind, amount, and relationship with the abuser) were considered. Out of a total of 196 children, 185 (95 females and 90 males) were included in the study. Two children diagnosed with autism spectrum disorder and 9 children with incomplete data regarding abuse were excluded from the data analyses. Three groups based on age were formed: 60 preschoolers (age range 2–6 years), 99 preadolescents (age range 7–12 years), and 26 adolescents (age range 13–18 years). With respect to the primary type of abuse, 40 (22%) were referred for sexual abuse, 70 (38%) for psychological abused, 27 (14%) for physical abuse, and 48 (26%) for neglect. However, about 20% of the youth who experienced sexual abuse had also substantiated histories of having been physically abused in the past.

Children experienced sexual abuse that involved sexual contact, noncontact sexual exposure, or both types of experiences. Sexual contact was defined as physical contact, such as kissing, fondling, or oral, anal, or vaginal stimulation or penetration. Noncontact exposure included exposure to pornographic materials, pornography of the child, exhibitionism, or having the child remove his or her clothes. Physical abuse and excessive physical punishment are defined broadly as any act committed by a parent or another person acting in a caregiving role that results in physical injury or threatened injury to a child (<18 years old), such as having been hit by a hand or object or having been kicked, shaken, thrown, burned, stabbed, or choked [34]. This abuse was documented by an independent party, such as a professional at the local social services, medical professional, mental health professional, or courts or by parents through an interview process.

Moreover, 120 participants (65%) had been abused by a family member and 65 (35%) by someone outside the family; 153 (83%) had suffered repeated maltreatment. Finally, 58 participants (31%) reported family history of psychiatric disorders.

### 2.2. Measures

Psychopathological diagnoses were established according to the* Diagnostic and Statistical Manual of Mental Disorders*, fourth edition (DSM-IV-TR) criteria [33]. Four categories of psychopathological disorders have been considered: internalizing disorders (anxiety and depressive disorders), externalizing disorder (attention deficit/hyperactivity disorder (ADHD), oppositional-defiant and conduct disorders), PTSD, and subclinical PTSD (sPTSD). The diagnosis of PTSD was made in preschoolers following the algorithm proposed by Scheeringa et al. [35]: two cluster A symptoms (exposure to trauma event) as for adults, one cluster B (reexperiencing/painful recall) symptom as for adults, one cluster C (avoidance/numbing) symptom instead of three or more as for adults, and two cluster D (hyperarousal) symptoms as for adults. For preadolescents and adolescents the diagnosis of PTSD was determined according to adult criteria. However, sPTSD was defined as a syndrome characterized by at least one symptom in each of three clusters: cluster B (reexperiencing/painful recall), cluster C (avoidance/numbing), and cluster D (hyperarousal) [36]. Psychiatric and psychopathological diagnoses were made by experienced developmental psychiatrists and neuropsychologists based on developmental history and extensive clinical examination. A medical history was obtained for all children who were also submitted to neurological and physical examinations. Moreover, in order to determine whether there were any familial psychiatric disorders, a detailed family history was taken for each patient by interviewing both parents. Family relationships were also evaluated by clinical observing sessions including all family members. Psychopathological assessment was conducted differently depending on children's age. In preschoolers, clinical observations of children's behavior were made during play sessions. Parents filled out the Child Behavior Checklist 1.5–5 [37], a parent-report questionnaire used to assess psychological and behavioral problems, and the Trauma Symptom Checklist for Young Children (TSCYC) [38], which provides a specific evaluation of PTSD. In the school-aged group (preadolescents and adolescents), the Schedule for Affective Disorders and Schizophrenia for School-Age Children-Present and Lifetime Version (K-SADS-PL) [39] was adopted; it is a semistructured interview designed for the assessment of current developmental psychopathology based on the DSM-IV-TR criteria. Parents filled out the CBCL 6–18 [37] and the Trauma Symptom Checklist for Children [40], which assesses PTSD. The children completed the Children Depression Inventory (CDI) [41] and the Multidimensional Anxiety Scale for Children (MASC) [42], two self-report questionnaires for a specific evaluation of mood states (depression and anxiety, resp.).

The study was conducted in accordance with the Declaration of Helsinki. Written informed consent was obtained from the parents of all participants.

### 2.3. Statistical Analysis

A series of *X* ^2^ analyses was performed on the prevalence (frequency) of different forms of maltreatment (sexual abuse, physical abuse, emotional abuse, and neglect) and of psychiatric disorders (sPTSD, PTSD, internalizing disorders, externalizing disorders, and no psychiatric disorders) within and between age groups (preschoolers, preadolescents, and adolescents) and within and between females and males. *p* values were adjusted by stepwise correction. A *t*-test was conducted to verify whether the age of maltreatment differed between families with history of psychiatric disorders and families with no history of psychiatric disorders. A *p* value of ≤.05 was considered significant.

## 3. Results

Our results documented that 51.3% of the participants displayed a sPTSD, 24.3% an internalizing disorder, 11.9% an externalizing disorder, and 7.6% a PTSD and 4.9% did not show any psychiatric disorders.

Significant differences emerged in the distribution of psychiatric disorders in the different forms associated with maltreatment considered [*X* ^2^(12) = 20.8, *p* = .05]. The sPTSD (see Table 1) was the most prevalent current psychopathological problem of each form of maltreatment. Compared to other forms of maltreatment, sPTSD was significantly more frequent in participants who have suffered sexual abuse, emotional abuse, physical abuse, and neglect (for the statistical comparisons see Supplementary Material available online at http://dx.doi.org/10.1155/2016/8654169, “Statistical Comparisons for Table  1”).

The distribution of forms of maltreatment differed between age groups, *X* ^2^(6) = 17.1, *p* < .009. In particular (see Table 2), in preschoolers physical abuse was less frequent than the other forms of maltreatment. In adolescents, the distribution of different forms of maltreatment did not differ. When we compared the three age groups in different forms of maltreatment, we found that in preadolescents emotional abuse was more frequent than in the two other groups. Moreover, sexual abuse was more frequent in preschoolers than in adolescents (for the statistical comparisons see Supplementary Material, “Statistical Comparisons for Table  2”).

The distribution of psychiatric disorders among age groups did not differ [*X* ^2^(8) = 13.9, *p* = .08]. A gender analysis was also conducted and a significant difference emerged [*X* ^2^(3) = 10.02, *p* = .019] (see Table 3). While, in males, the most frequent form of maltreatment was emotional abuse, in females, physical abuse was less frequent than the other forms. When directly comparing females with males in forms of maltreatment, it emerged that sexual abuse was more frequent in females than in males (for the statistical comparisons see Supplementary Material, “Statistical Comparisons for Table  3”). Moreover, in females as well in males (see Table 4), sPTSD was the most frequent psychiatric disorder. When directly comparing female participants with male participants in psychiatric disorders, it was found that only externalizing disorders were more frequent in males than females (for the statistical comparisons see Supplementary Material, “Statistical Comparisons for Table  4”).

Finally, with regard to the history of psychiatric disorders, data showed that age at time of maltreatment in children with family history of psychiatric disorders (mean = 5.82, sd = 2.96) was lower [*t*(183) = 2.72, *p* = .001] than the age of children without family history of psychiatric disorders (mean = 7.41, sd = 3.46). However, the distribution of different types of maltreatment, of psychiatric disorders, and of gender did not differ between children with or without family history of psychiatric disorders (*p* always > .10).

## 4. Discussion

Our study explores the current psychopathological problems in maltreated children assessed by a clinical examination. Results documented that more than half of children and adolescents did not meet the full criteria for a diagnosis of psychopathological disorder. However, the distribution of sPTSD was found to be the most prevalent current psychopathological problems associated with maltreatment. Our results also showed that the incidence of PTSD in children after exposure to a traumatic event is lower than that reported in adults [43, 44].

A possible interpretation for this discrepancy is that PTSD diagnosis derived from criteria developed for adults (DSM-IV-TR) [33] and that these criteria may not accurately reflect responses to trauma in children. Indeed, PTSD criteria do not capture some reactions of infants and children, who suffered different forms of abuse, often experienced as developmental delays, complex disruption of affect regulation, disturbed attachment patterns, rapid behavioral regressions, and emotional shifting [45]. Accordingly, studies indicate low reliability of PTSD symptoms in children [46, 47] and low diagnostic efficacy of arousal symptoms [48]. Analytic studies in children have often failed to support the 3-symptom clusters of painful recall, arousal, and avoidance described in the adult literature [49, 50] and researches on children suggest that the optimal algorithm for PTSD may require substantially fewer symptoms than those required to diagnose this disorder in adults [27, 51–53]. However, subclinical signs should not be overlooked and therapeutic actions should be taken in children since long-term negative outcomes have been documented, such as psychological and behavioral problems, psychiatric and somatic disorders, and neurocognitive deficits [18, 54].

Two models have influenced the research so far: [55]: the* general effects model* which suggests that all types of maltreatment are traumatizing and that any childhood trauma leads to impairment in psychological functioning [56, 57] and the* differential effects model* which holds that specific types of child maltreatment are associated with specific outcomes [56, 57].

Results from the present study, documenting a broad spectrum of effects, support the general effects model on child maltreatment outcomes. Indeed, emotional and behavioral dysfunctions following child maltreatment do not vary depending on different forms of maltreatment suffered.

A further result, from our study, is that forms of maltreatment differed by age and gender. While in adolescents the distribution of forms of maltreatment did not change, in preadolescents emotional abuse was more prevalent than the other forms of maltreatment and than in the other two age groups. Data from literature documented a different distribution of type of maltreatment by age. Epidemiological studies showed that adolescents (12–14 years) suffer more sexual abuse than other age groups while the preschoolers suffer more neglect and medical neglect than other forms of maltreatment [58]. Nonetheless, previous findings were not directly comparable with our data since the existing studies referred to statistical reports from general population while we recruited children and families who reached our unit for suspected maltreatment. Jud et al. [59] analyzed the distribution of different types of maltreatment in children who referred to a child protection hospital team for suspected abuse. Their results documented that the mean age of the maltreated children ranges from 5 years in those neglected to 8.4 years in those sexually abused. Compared to the mean age of 7 years in physically maltreated children, younger children are at significantly higher risk of being neglected while older children are at a significantly higher risk of being sexually maltreated.

Concerning the effects of maltreatment, evidences provided results on psychopathological effect within age groups while a comparison of the effects between different age groups is still lacking. Studies on preschoolers' exposure to violence (including sexual abuse and physical abuse) found a significant association with both internalizing and externalizing symptoms [60, 61] whereas studies on preadolescents experiencing physical or sexual abuse revealed high risk for externalizing behavior [62]. Moreover, preadolescents suffering physical abuse and/or physical neglect were more likely to have higher levels of caregiver-reported internalizing problems [62]. Results from the present study failed to describe differences in the prevalence of psychopathological disorders among the three age groups.

As regards gender, our intragroup analysis showed that in males the most frequent form of maltreatment was emotional abuse. From the intergroup comparison, sexual abuse was found more prevalent in females than in males. This result fits in with a recent study of prevalence from a population survey of child maltreatment and other types of victimization in the UK [63], where sexual abuse was found more frequent in females. However, males experienced more victimization by peers, more physical violence from noncaregivers, and more exposure to community violence. The distribution of psychiatric disorders in males and females did not differ in our study and sPTSD was found to be the most current psychopathological problem associated with maltreatment in both groups. From intergroup comparisons it emerged that externalizing disorders were more frequent in males than females. This finding is consistent with existing studies showing that in stressful situations boys tend to express anger and act out aggressively while girls are more likely to cope by internalizing their response [64, 65]. More specifically, a recent study [66] on early child maltreatment documented, among boys, a mean score of externalizing problems almost five points higher than controls while, among girls, no significant differences between internalizing and externalizing problems but became stronger over the course of the follow-up examinations [66].

Finally, we found that children who grow up in families with a psychiatric disorder history were exposed to abuse earlier (about one and a half years younger). Furthermore, children with or without family history of psychiatric disorders did not differ in the distribution of types of maltreatment, psychiatric disorders, and gender. It has been demonstrated that the risk for physical abuse is linked to some parenting characteristics as low engagement and negative perceptions of the child [67]. The prevalent exposure to potential traumatic events is significantly associated with contextual risks in children's lives, such as living in a single-parent home, in high parenting stress, and particularly with clinical levels of parental mood and anxiety symptoms [60].

In conclusion, our study revealed that the most common current psychopathological problem in children and adolescents who have suffered different forms of maltreatment was a subclinical form of PTSD. The construct of DESNOS (“disorders of extreme stress not otherwise specified”) represents an attempt to capture the multidimensional nature of the failure of adaptation to the trauma although it has yet to be determined systematically [68]. Indeed, children develop a range of changing maladaptive patterns, which depend on their stage of development, social support, and relationship with the source of the trauma [68]. According to the difficulty in capturing children's reactions with current PTSD criteria, DSM 5 [69] reports a new classification regarding “Trauma and Stressors Related Disorder” which includes specific diagnoses for children under 6 years of age. With respect to current psychiatric classifications, if we consider the negative long-term effects associated with maltreatment our study emphasizes the importance of early diagnosis and treatment. Accordingly, we stress the need for longitudinal studies to follow up the long-term effects on mental health by taking into account also individual and familial characteristics (including socioeconomic status).

Future research will focus on the possibility of more detailed analysis of current neuropsychological problems in the context of maltreatment (including memory, executive functions, working memory, and attention) and maltreatment characteristics (including duration and intensity of exposure).

Another limitation of the study is the exclusion of children with developmental disorders with severe functional impairment (as children with autism spectrum disorder) because of incomplete data regarding abuse and because of avoidance of implying that any maltreatment had caused the autism. Future research will analyze psychopathological problems associated with maltreatment also in the group of children with defined developmental disorders as well as children with autism spectrum disorder or with attention deficit hyperactivity disorder.

## Supplementary Material

Statistical Comparisons for Table 1: Compared in any forms of maltreatment sPTSD respect to the other Psychiatric Disorder and not Psychiatric Disorder. Statistical Comparisons for Table 2: Compared in any age range the distribution of the different forms of maltreatment and then compared the distribution of the different forms of maltreatment between the three age rage. Statistical Comparisons for Table 3: Compared for each gender the distribution of the different forms of maltreatment and then compared the distribution of the different forms of maltreatment between genders. Statistical Comparisons for Table 4: Compared for each gender the distribution of the different Psychiatric Disorders and then compared the distribution of the different Psychiatric Disorders between genders.

## Figures and Tables

**Table 1:** Distribution of psychiatric disorders in the different forms associated with maltreatment.

	Form of maltreatment				*N*
	Sexual	Emotional	Physical	Neglect	
Psychiatric disorders					
No psychiatric disorders	2 (22.2%)	4 (44.4%)	1 (11.1%)	2 (22.2%)	9
sPTSD	18 (18.9%)	39 (41.1%)	13 (13.8%)	25 (26.3%)	95
PTSD	8 (57.1%)	4 (28.6%)	2 (14.3%)	—	14
Internalizing disorders	9 (20%)	17 (37.8%)	4 (8.9%)	15 (33.3%)	45
Externalizing disorders	3 (13.6%)	6 (27.3%)	7 (31.8%)	6 (27.3%)	22

*N*	40	70	27	48	185

**Table 2:** Distribution of the different forms of maltreatment within and between age groups.

	Form of maltreatment				*X* ^2^
	Sexual	Emotional	Physical	Neglect	
Age					
Preschoolers	20 (33.3%)	22 (36.7%)	5 (8.3%)	13 (21.7%)	11.9^*∗∗*^
Preadolescents	13 (13.1%)	44 (44.4%)	15 (15.2%)	27 (27.3%)	24.6^*∗∗∗*^
Adolescents	7 (27%)	4 (15%)	7 (27%)	8 (31%)	1.04

*X* ^2^	6.3^*∗*^	34.4^*∗∗∗*^	6.2^*∗*^	12.1^*∗∗*^	

*Note*. After stepwise correction: *p* < .05^*∗*^, *p* < .01^*∗∗*^, and *p* < .001^*∗∗∗*^.

**Table 3:** Distribution of the different types of maltreatment in male and female participants.

	Form of maltreatment				*X* ^2^
	Sexual	Emotional	Physical	Neglect	
Gender					
Males	13 (14.4%)	39 (43.3%)	18 (20%)	20 (22.2)	17.3^*∗∗∗*^
Females	27 (28.4%)	31 (32.6%)	9 (9.5%)	28 (29.5)	12.6^*∗∗*^

*X* ^2^	4.9^*∗*^	0.9	3	1.3	

*Note*. After stepwise correction: *p* < .05^*∗*^, *p* < .01^*∗∗*^, and *p* < .001^*∗∗∗*^.

**Table 4:** Distribution of psychiatric disorders in the gender groups.

	Psychiatric disorder					*X* ^2^
	No psychiatric disorders	sPTSD	PTSD	Internalizing disorders	Externalizing disorders	
Gender						
Males	3 (3.3%)	41 (45.6%)	4 (4.4%)	24 (26.7%)	18 (20%)	54.8^*∗∗∗*^
Females	6 (6.3%)	5 (56.8%)	10 (10.5%)	21 (22.1%)	4 (4.2%)	62.5^*∗∗∗*^

*X* ^2^	1	0.09	2.6	0.02	8.9^*∗∗*^	

*Note*. After stepwise correction: *p* < .01^*∗∗*^, and  *p* < .001^*∗∗∗*^.

## Additional Points

The aim of the present study is to evaluate the current psychopathological problems of different forms associated with maltreatment on children's and adolescents' mental health. Results documented that 95.1% of abused children and adolescents developed a psychiatric disorder or a subclinical form of a Posttraumatic Stress Disorder (PTSD). Moreover, our data demonstrate a role for gender, age, and familial psychiatric comorbidity in current psychopathological problems associated with maltreatment. We stress the need for longitudinal studies to follow up the long-term effects on mental health by taking into account also individual and familial characteristics (including socioeconomic status). Future research will focus on the possibility of analyzing more in detail the current psychopathological problems associated with maltreatment regarding neuropsychological assessment (including memory, executive functions, working memory, and attention) and maltreatment characteristics (including duration and intensity of exposure).
