# Supplementary material for: Current Psychopathological Symptoms in Children and Adolescents Who Suffered Different Forms of Maltreatment
Source: ScientificWorldJournal. 2016 Aug 4;2016:8654169. doi: 10.1155/2016/8654169 (PMC4989087; doi:10.1155/2016/8654169)
Supplement: Supplementary file 1 — Statistical Comparisons for Table 1: Compared in any forms of maltreatment sPTSD respect to the other Psychiatric Disorder and not Psychiatric Disorder. Statistical Comparisons for Table 2: Compared in any age range the distribution of the different forms of maltreatment and then compared the distribution of the different forms of maltreatment between the three age rage. Statistical Comparisons for Table 3: Compared for each gender the distribution of the different forms of maltreatment and then compared the distribution of the different forms of maltreatment between genders. Statistical Comparisons for Table 4: Compared for each gender the distribution of the different Psychiatric Disorders and then compared the distribution of the different Psychiatric Disorders between genders. [file 8654169.f1.pdf]

Supplementary materials

Statistical comparisons for Table 1

sPTSD *vs* No Psychiatric Disorders: $X^2(1) = 12.8$, $p < .001$; sPTSD *vs* PTSD: $X^2(1) = 3.85$, $p < .05$; sPTSD *vs* Externalizing Disorders: $X^2(1) = 10.71$, $p < .001$], Emotional Abuse [sPTSD *vs* No Psychiatric Disorders: $X^2(1) = 28.49$, $p < .001$; sPTSD *vs* PTSD: $X^2(1) = 28.49$, $p < .001$; sPTSD *vs* Externalizing Disorders: $X^2(1) = 24.2$, $p < .001$], Physical Abuse [sPTSD vs No Psychiatric Disorders *vs*: $X^2(1) = 10.29$, $p < .01$; sPTSD *vs* PTSD: $X^2(1) = 8.06$, $p < .01$; sPTSD *vs* Internalizing: $X^2(1) = 4.77$, $p < .05$] and Neglect [sPTSD *vs* No Psychiatric Disorders: $X^2(1) = 19.59$, $p < .001$; sPTSD *vs* PTSD: $X^2(1) = 25$, $p < .001$; sPTSD *vs* Externalizing: $X^2(1) = 11.65$, $p < .001$].

Statistical comparisons for Table 2

Physical vs Sexual: X2(1) = 9, p < .01; Physical vs Emotional: X2 (1) = 10.7, p < .01; Physical vs Neglect; X2(1) = 3.6, p < .05]. However, in preadolescents Emotional Abuse was more frequent than the other forms of maltreatment [Emotional vs Sexual: X2(1) = 16.9, p < .001; Emotional vs Physical: X2(1) =14.3, p < .001; Emotional vs Neglect: X2(1) = 4.1, p < .05.

In adolescents, the distribution of different forms of maltreatment did not differ [$X^2(3) = 1.4$, $p = .7$].

In preadolescents, Emotional Abuse was more frequent than in the two other groups [preadolescents *vs* preschoolers $X^2(1) = 7.3$; $p < .01$; preadolescents *vs* adolescents $X^2(1) = 33.3$; $p < .01$].

Sexual Abuse was more frequent in preschoolers than in adolescents [$X^2(1) = 6.3$; $p < .01$].

Statistical comparisons for Table 3

The most frequent form of maltreatment in males was Emotional Abuse [Emotional *vs* Sexual: $X^2(1) = 13$, $p < .01$; Emotional *vs* Physical: $X^2(1) = 7.7$, $p < .01$; Emotional *vs* Neglect: $X^2(1) = 6.1$, $p < .01$]

In females, Physical Abuse was less frequent than the other forms [Physical *vs* Sexual: $X^2(1) = 9$, $p < .01$; Physical *vs* physical: $X^2(1) = 12.1$, $p < .01$; Physical *vs* Neglect: $X^2(1) = 9.8$, $p < .01$].

Sexual Abuse was more frequent in females than in males [$X^2(1) = 4.9$; $p < .01$].

Statistical comparisons for Table 4

In males sPTSD was more frequent than the other forms of psychiatric disorder [sPTSD *vs* No Psychiatric Disorders: $X^2(1) = 32.8$, $p < .001$; sPTSD *vs* PTSD: $X^2(1) = 30.4$, $p < .001$; sPTSD *vs* Internalizing Disorders: $X^2(1) = 4.4$, $p < .05$; sPTSD *vs* Externalizing Disorders: $X^2(1) = 9$, $p < .01$]. Moreover, both Internalizing and Externalizing Disorders were more prevalent than PTSD [Internalizing Disorders *vs* PTSD: $X^2(1) = 14.3$, $p < .001$; Externalizing Disorders *vs* PTSD: $X^2(1) = 9$, $p < .01$] and than No Psychiatric Disorders [Internalizing Disorders *vs* No Psychiatric Disorders: $X^2(1) = 16.3$, $p < .001$; Externalizing Disorders *vs* No Psychiatric Disorders: $X^2(1) = 10.8$, $p < .001$].

In females as well in males, sPTSD was the most frequent psychiatric disorder [sPTSD *vs* No psychiatric disorders: $X^2(1) = 38.4$, $p < .001$; sPTSD *vs* PTSD: $X^2(1) = 31$, $p < .001$; sPTSD *vs* Internalizing Disorders: $X^2(1) = 14.5$, $p < .001$; sPTSD *vs* Externalizing Disorders: $X^2(1) = 43.1$, $p < .001$].

In females Internalizing Disorder was more prevalent than PTSD [$X^2(1) = 4$, $p < .05$], than Externalizing Disorders [$X^2(1) = 11.6$, $p < .001$] and than No Psychiatric Disorders [$X^2(1) = 8.3$, $p < .01$].

Externalizing Disorders were more frequent in males than females [$X^2 (1) = 8.9$, $p < .01$].
